# Travel Mode Detection with Varying Smartphone Data Collection Frequencies

**DOI:** 10.3390/s16050716

**Published:** 2016-05-18

**Authors:** Muhammad Awais Shafique, Eiji Hato

**Affiliations:** 1 Department of Transportation Engineering and Management, University of Engineering and Technology, GT Road, Lahore 54890, Pakistan; 2 Transportation Research and Infrastructure Planning Laboratory, Department of Civil Engineering, The University of Tokyo, 7-3-1, Hongo, Bunkyo-ku, Tokyo 113-8656, Japan; hato@bin.t.u-tokyo.ac.jp

**Keywords:** classification, moving window, random forest, smartphone, travel mode

## Abstract

Smartphones are becoming increasingly popular day-by-day. Modern smartphones are more than just calling devices. They incorporate a number of high-end sensors that provide many new dimensions to smartphone experience. The use of smartphones, however, can be extended from the usual telecommunication field to applications in other specialized fields including transportation. Sensors embedded in the smartphones like GPS, accelerometer and gyroscope can collect data passively, which in turn can be processed to infer the travel mode of the smartphone user. This will solve most of the shortcomings associated with conventional travel survey methods including biased response, no response, erroneous time recording, *etc.* The current study uses the sensors’ data collected by smartphones to extract nine features for classification. Variables including data frequency, moving window size and proportion of data to be used for training, are dealt with to achieve better results. Random forest is used to classify the smartphone data among six modes. An overall accuracy of 99.96% is achieved, with no mode less than 99.8% for data collected at 10 Hz frequency. The accuracy is observed to decrease with decrease in data frequency, but at the same time the computation time also decreases.

## 1. Introduction

Household trip data are of crucial importance for managing present transportation infrastructure as well as to plan and design future facilities. They also provide basis for new policies implemented under Transportation Demand Management (TDM). The methods used for household trip data collection have changed with passage of time, starting with the conventional face-to-face interviews or paper-and-pencil interviews in the 1950s. High cost and safety issues proved to be the major problems in this approach. To overcome such disadvantages, computer assisted surveys were introduced in the 1980s. These surveys included computer-assisted telephone interview (CATI) and computer-assisted self-interview (CASI) [1,2]. The computer assisted surveys proved to be an improvement from the previous face-to-face interviews [3] but the underlying shortcomings in person trip (PT) data collection methods still remained. These included inaccuracies in recording the starting and ending times, underreporting due to missing short trips and non-response [4,5]. The source of all these problems was the enormous burden on the respondents to answer a huge number of questions based on their memories. To address this issue, GPS technology was employed during the late 1990s, providing the starting point for a generation of smart travel survey methods [6]. 

Initially, GPS surveys were carried out as supplementary surveys to assess the accuracy of traditional methods, but later total replacement was experimented with [7,8,9]. At the beginning, GPS devices were installed in vehicles. Consequently, only the travel behavior of people using vehicles was monitored. In the early 2000s, rapid advancement in technology paved way for the development of wearable GPS data loggers [10]. With the introduction of lightweight, portable and handy GPS data loggers, all modes of transportation could be monitored. Although GPS devices can very accurately record the locations and time-stamps, important information like travel mode and trip purpose are not recorded. These details are inferred from the GPS data by appropriate data processing [11]. 

Recently, the explosive spread of smartphones has provided the transportation community with a new potential and a lot of research is being carried out to utilize smartphones for travel data collection. This interest is because of GPS sensors being embedded into modern smartphones, making it possible to replace the GPS data loggers being used previously. Smartphones have an added advantage of being a necessary travel companion, hence being able to monitor the travel patterns over extended periods of time. Recently, GPS enabled smartphones are also utilized for indoor positioning and pedestrian navigation [12,13,14]. On the other hand, GPS loggers are considered a burden to carry around. The inclusion of accelerometer in smartphones has dramatically enhanced its capability to accurately detect the travel mode and trip purpose. Accelerometer can detect accelerations along three axes (x, y and z) with respect to the gravitational force. It means that at rest, the accelerometer will register an acceleration of 9.8 m/s^2^ along the downward direction. Orientation augments the accelerometer data by providing the information regarding angular motion. Orientation sensor is software-based and drives its data from the accelerometer and the geomagnetic field sensor. The current study focuses on the development of data-processing methodology for travel mode detection using accelerometer and orientation data collected by smartphones.

GPS devices have been used by many researchers for the purpose of mode detection, whether employing rule-based algorithms [15,16,17,18], or machine learning algorithms [19,20,21]. 

Before smartphones came to the spotlight, the possibility of utilizing mobile phones for data collection using GSM technology was explored [22]. Rather than employing GPS, locations were derived from mobile communication towers to be used for reconstructing travel patterns [23]. Soon, more technology solutions were explored including Bluetooth, WiFi, RFID and smart-cards [1]. Personal handy phone systems (PHS) became very popular in Japan for recording geographical locations. These systems located the device with the help of base stations [24,25]. Over 20 case studies have been conducted in Japan using PHS since 2003 [26,27,28]. 

The tremendous popularity and increasing penetration of smartphones has attracted much research attention on their role in identifying the mode of transportation [29,30,31,32,33]. Most of the studies have a similar methodology where suitable features were extracted from the raw sensor data, a training dataset was used to train a classification algorithm and then the algorithm was used to predict the test data based on the heuristics learned during the training phase. Transportation mode identification accuracy increased when GPS data were linked to GIS platform [34]. The accuracy was further improved by combining GPS and accelerometer data for mode detection [35].

A study by Tsui and Shalaby [36] collected GPS data from Toronto. Accelerations, average and maximum speeds extracted from the GPS data along with public transportation route information, was used to predict the transportation modes, achieving a prediction accuracy of more than 90%. Another study performed in the same area used one participant to replicate 60 trips recorded during the ‘Toronto Transportation Tomorrow Survey’, carrying a GPS device [37]. After collecting the GPS data and combining it with the GIS information available, a mode prediction accuracy of 92% was achieved. Another study extracted features like average accuracy of the GPS coordinates, average speed, average heading change, average acceleration, bus location proximity, rail line trajectory proximity, bus stop proximity rate and zip code, using collected GPS data accompanied by ground conditions [30]. Five different classification algorithms were tested, with results suggesting that random forest outperforms others. A study named Future Mobility Survey (FMS) by Pereira [38] compared the traditional survey results with survey by smartphones. It is part of a research project initiated by an alliance between Singapore and Massachusetts Institute of Technology (MIT). The study validated that participants tend to over-estimate the travel time in traditional surveys. 

Smartphones are equipped with a range of sensors, many of which are not favored by or simply overlooked by majority of researchers. However, there are some studies that incorporate these sensors as well. Frendberg [39] utilized data collected from GPS, accelerometer, orientation sensor and magnetic sensor to detect the travel mode using a smartphone application, similar to Su and Caceres [40].

A number of studies have utilized the accelerometer data alone for classification purposes [41,42,43,44,45,46]. In one study [29], the training and testing datasets were formed by taking 70% of the collected data as training data and rest as test data; a similar study divided the collected data as 90% for training and 10% for testing [47]; and yet another study used almost 50% of the collected data for training and rest for testing the classification algorithms [48]. Some studies (e.g., [49]) also collected GPS data but for data validation only. Mode detection was still managed by accelerometer data.

Various studies have compared random forest with other algorithms for the purpose of mode detection, while reaching the same conclusion that random forest is a superior algorithm for the intended purpose. For instance, one study made a comparison among random forest, naïve Bayes, Bayesian network, decision trees and multilayer perceptron [30]; another incorporated neural network and support vector machines along with random forest [32]; one more studied random forest, k-nearest neighbor, support vector machines, naïve Bayes and decision trees [21]; and further a study reported a comparison among support vector machines, adaptive boosting, decision trees and random forest [50]. These studies demonstrated that random forest yields higher travel mode prediction accuracies. 

In our previous studies [50,51], acceleration data were collected by a purpose-built wearable device named as BCALs (Behavioral Context Addressable Loggers in the Shell). Mode detection was successfully done among four modes: walk, bicycle, car and train. Developing a methodology for data collected by smartphones and also to add some other modes for classification was required. Therefore, our current work proposes a methodology for identification among six different travel modes namely walk, bicycle, car, bus, train and subway, using data from accelerometer and orientation sensors embedded in smartphones. Further, it investigates the effect of various data collection frequencies on the classification accuracy of the used algorithm as well as the computational costs incurred.

## 2. Method 

### 2.1. Data Collection

**Figure 1:**
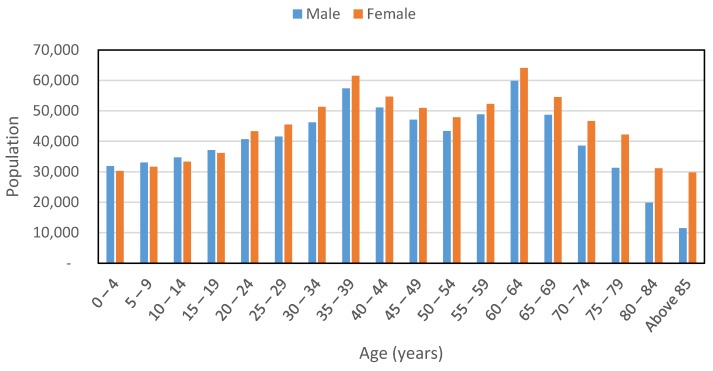
Demographics of Kobe city.

Fifty participants from Kobe, Japan contributed to the collection of data utilizing Android smartphones, over a month during November 2013. The data collection days varied among the participants, with some providing the records for only single day travel, while others cooperating for multiple days. Consequently, the collected data are quite few as compared to one-month collection time. Six modes were observed, *i.e.*, walk, bicycle, car, bus, train and subway. Recording of the ground truth was achieved by a simple application installed in the smartphones. The participants would merely input the travel mode in the application while starting a trip, and then stop the recording once they have reached their destination. At the end of the day, a recall survey would be conducted to check the reliability of the collected data. With the help of route maps generated by the GPS data, the participants could easily reconfirm the starting and ending times of various trips as well as the mode of transportation used. Afterwards, only the sensor data associated with the trips were retained and all other data including any problematic data or unlabeled data were discarded (Table 1). The distribution of participants according to gender as well as age is shown in Table 2. Although the participants’ demographics are not used in the analysis, it is worth mentioning because it implicitly affects the collected data. Table 2 shows that almost all age groups capable of driving and using other modes of transportation are incorporated in this study. Demographic data were collected during several meetings, where the participants were enrolled in the program. The general demographics of Kobe, according to 2010 census, are presented in Figure 1. The participants do not strictly represent the general demographics, *i.e.*, male participants are more than female participants, as this was a limitation of the willingness of people to participate in the survey.

The collected data consisted of readings by accelerometer (accelerations along z-, y- and z-axes) and orientation sensor (pitch and roll). GPS data were also collected but were used in this study for data verification only. For mode detection, it was dropped as the aim was to devise a battery-efficient methodology. The sensors recorded data at an average frequency of 14 Hz but due to the varying frequencies among the users, the data collection frequency was scaled down to a uniform 10 Hz. Further decreased frequencies were also tested to compare them with respect to their computational costs (details in Section 2.6). An additional advantage of decreasing the frequency can be in making the procedure more battery-efficient, as battery time is one of the main obstacles in data collection using smartphones. This can be visualized by the power consumption figures provided in the literature [52]. The study reported that an accelerometer collecting the readings at 20 Hz frequency consumes 230 mW. The power consumption reduces to 180 mW for 10 Hz frequency, and further reduces to 164 mW for 2 Hz frequency. Unfortunately, during data collection for the current study, the battery usage was not tracked, making it impossible to carry out energy consumption analysis. Nevertheless, energy consumption is an issue when it comes to employing smartphones; therefore, it was partially dealt here by reducing the data collection frequency. Table 3 provides the number of trips and the amount of data instances recorded for each mode at 10 Hz frequency. The percentages do not add up to 100 because of rounding.

### 2.2. Pre-Processing

Smartphones are usually carried in different positions by the users, e.g., some place their smartphones in their pockets, some carry it in their purse and some simply keep it in their hands while messaging or calling. These different orientations make it difficult to individually use the accelerations along the coordinate axes because smartphones’ accelerometer record accelerations with respect to the force of gravity. Therefore different orientations affect the individual accelerations differently. To solve this problem, like some other studies [47,53,54,55], instead of using accelerations along the three axes individually, magnitude of the resultant acceleration was used, calculated as below.

(1) $$A_{res}=\sqrt{A_{x}{}^{2}+A_{y}{}^{2}+A_{z}{}^{2}}$$

**Figure 2:**
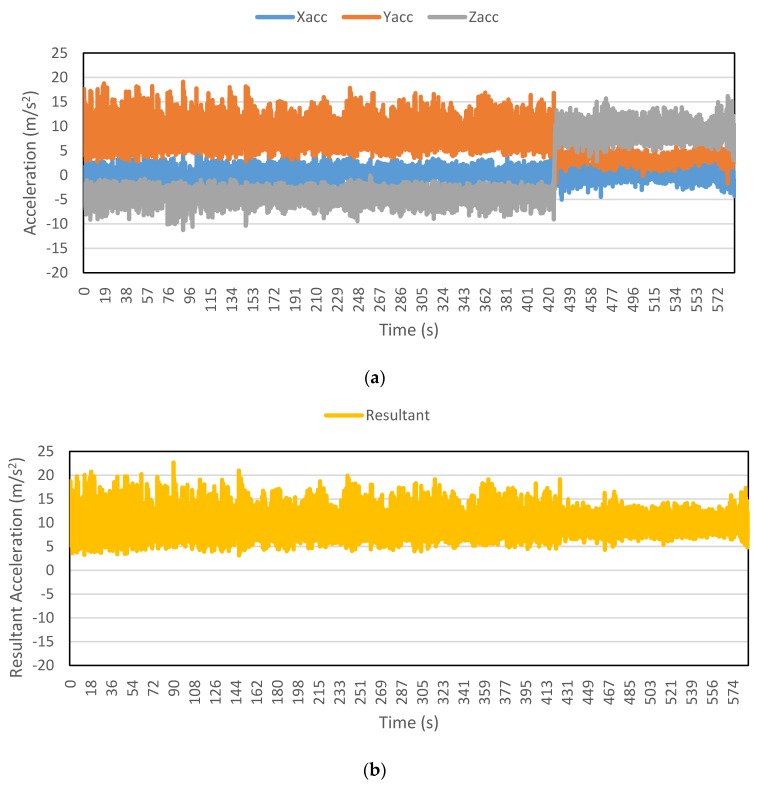
Accelerations recorded for a walk trip: (**a**) Accelerations along three axes; and (**b**) Resultant acceleration.

**Figure 3:**
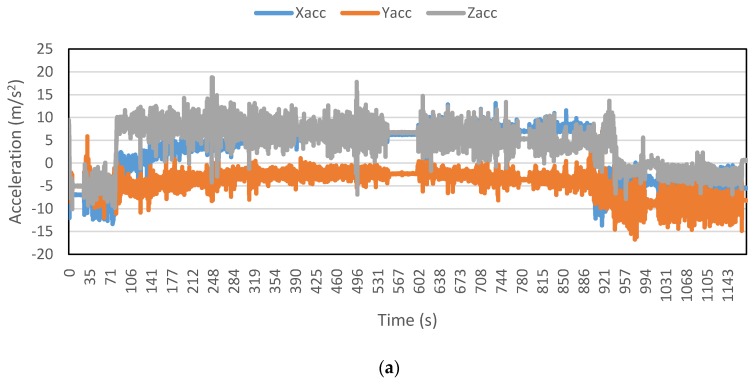

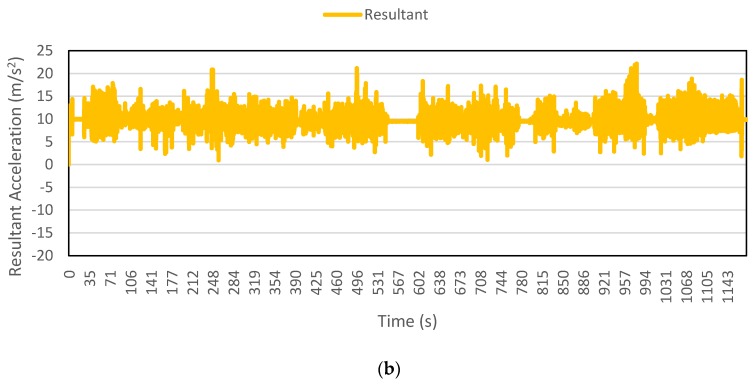
Accelerations recorded for a bicycle trip: (**a**) Accelerations along three axes; and (**b**) Resultant acceleration.

**Figure 4:**
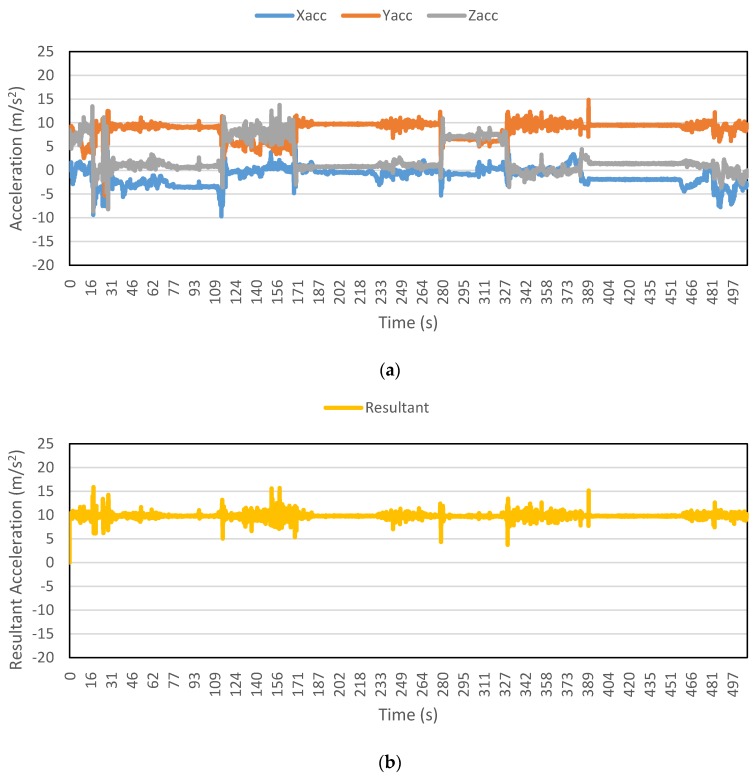
Accelerations recorded for a car trip: (**a**) Accelerations along three axes; and (**b**) Resultant acceleration.

**Figure 5:**
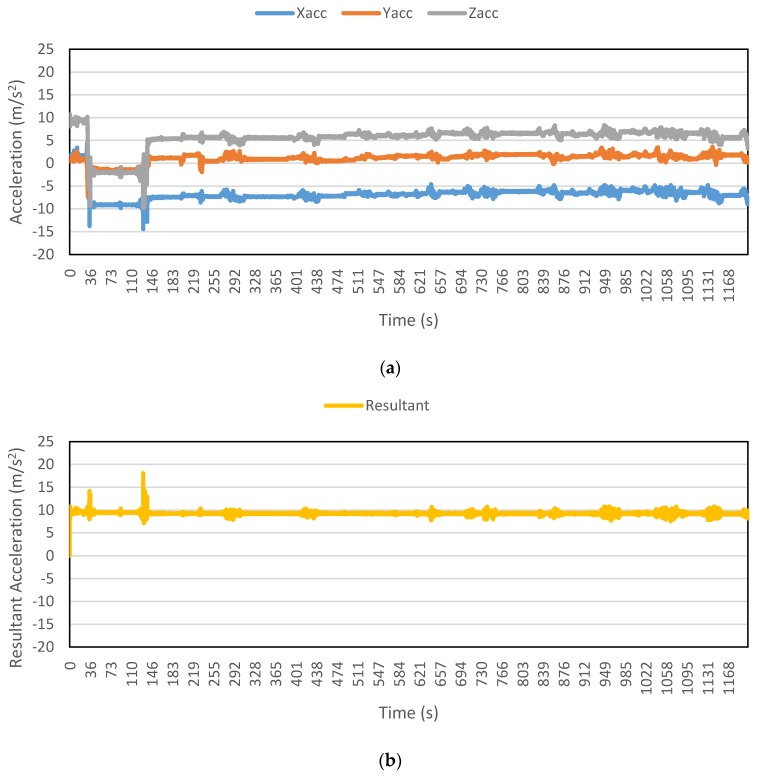
Accelerations recorded for a bus trip: (**a**) Accelerations along three axes; and (**b**) Resultant acceleration.

**Figure 6:**
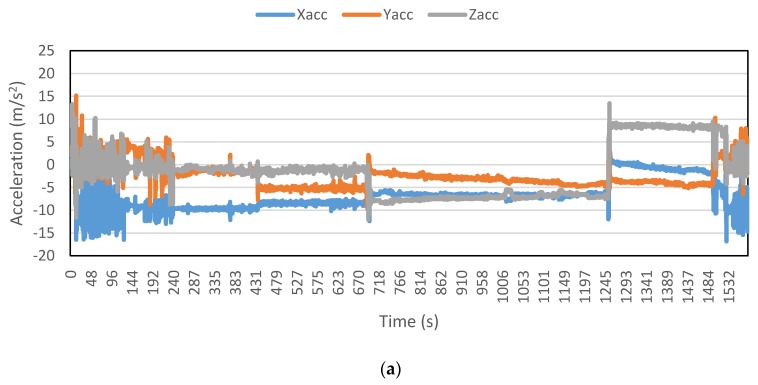

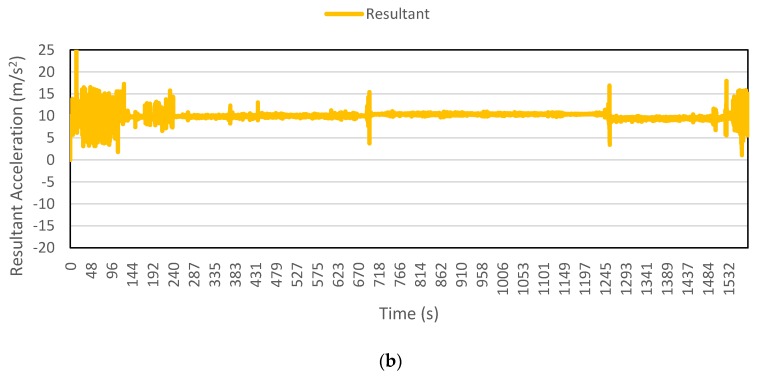
Accelerations recorded for a train trip: (**a**) Accelerations along three axes; and (**b**) Resultant acceleration.

**Figure 7:**
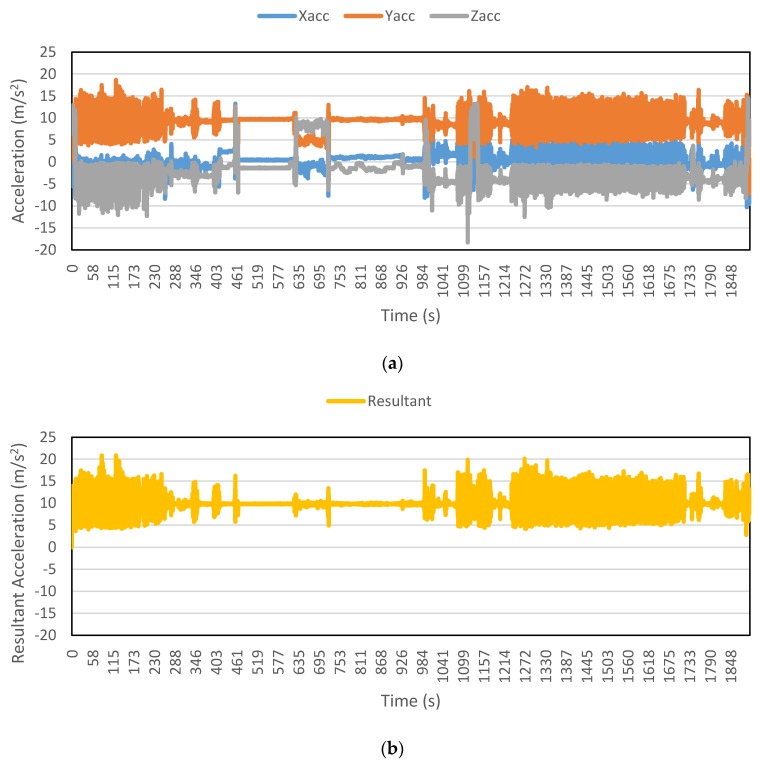
Accelerations recorded for a subway trip: (**a**) Accelerations along three axes; and (**b**) Resultant acceleration.

Figure 2, Figure 3, Figure 4, Figure 5, Figure 6 and Figure 7 exhibit the accelerations recorded along the three axes and their calculated resultant for each mode over a single trip respectively. The change in position of the smartphone can be observed by the abrupt shift in acceleration values in Figure 3a. This is the reason that individual accelerations were not used; instead, the magnitude of their resultant was utilized in the analysis. Furthermore, it is evident from the figures that the non-motorized modes, *i.e.*, walk and bicycle, register a lot of fluctuations, whereas the behavior of motorized modes is different, with comparatively smooth trends. It can also be noted that resultant acceleration alone is not sufficient to distinguish among the modes, therefore some other features need to be extracted (details in Section 2.3). The magnitude of resultant acceleration might be affected by the activity performed on the smartphone by the owner, like calling or texting or no action at all. This probable effect should be investigated further as mentioned in future work. 

Wolf [56] used a dwell time of 120 s for trip identification. The value was based on the design criteria mentioned in the Highway Capacity Manual, where the traffic signal cycle should be less than 120 s. It was assumed that stoppage at traffic signals should not be considered as trip ends. The 120 s rule lacked empirical results to support it [11]. Shen and Stopher [57] tested different thresholds of dwell time from 15 s to 120 s and concluded that 60 s would be a better criterion for trip segmentation. In the current study, the same dwell time of 60 s was used to identify different trips. In other words, if two consecutive readings were more than 60 s apart then they were considered as the ending point of the previous trip and starting point of the next trip, respectively. 

This simple solution was applicable because only the sensors’ data associated with the trips was taken; hence, various trips were already segmented as far as the data were concerned. It also resulted in identifying one trip as several independent trips due to short stops on the way, for instance waiting at intersections. This was not a serious issue as the only aim of the current study was to identify the mode of transportation. As long as the mode is detected correctly, it does not matter whether it is one trip or several trips. The process of splitting and joining of trips will be developed in future research. A much better methodology for stop detection is proposed by Xiao and Low [58], but it requires collection of GPS and GSM-based positioning data.

### 2.3. Feature Extraction

In addition to resultant acceleration, six features were further extracted from resultant acceleration namely standard deviation, skewness, kurtosis, maximum resultant acceleration, average resultant acceleration and maximum average resultant acceleration. Pitch and roll, directly recorded by orientation sensor, were also considered for classification. 

**Figure 8:**
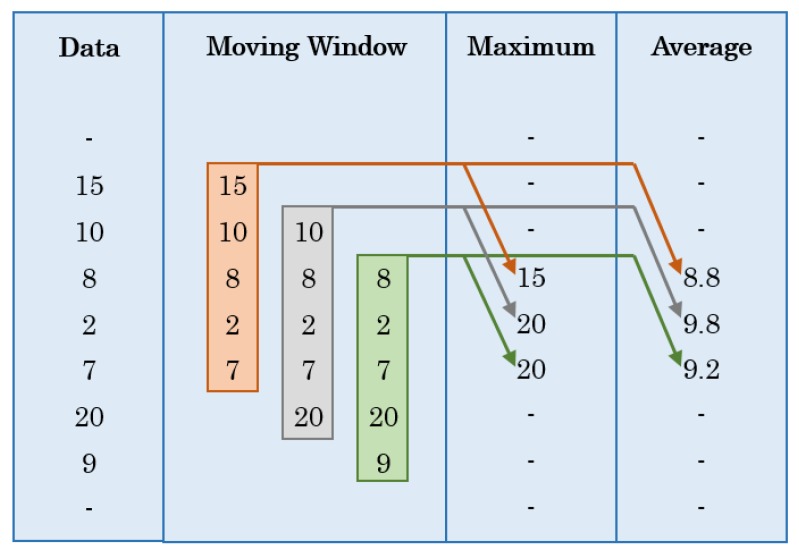
Application of the moving window concept.

Most of the extracted features are quite straightforward. Skewness measures the lack of symmetry of a given dataset. A dataset is symmetric if it looks the same on both sides of the center point. On the other hand, kurtosis measures the flatness of the dataset, determining whether the dataset or distribution is peaked or flat around the mean, relative to normal distribution. After average resultant accelerations were calculated, they were used to calculate maximum average resultant accelerations in the same way as resultant accelerations were used to calculate maximum resultant accelerations, over each window. All features/variables, except resultant acceleration, were calculated by employing a moving window concept [50]. For the purpose of smoothening the data and reducing the effect of the outliers, the concept of moving window was used where a certain number of readings, defined by the window size, were used to apply an operation (e.g., average, maximum, *etc.*) at a certain data entry level and this window moved downwards as the calculations proceeded along the data column. Suppose five data readings fall in 1 min window, then Figure 8 shows an example of how moving window concept is applied.

Although the window size was reported in the form of time, the equation developed for the calculation took into account the number of instances covered in the reported time interval. For example, a 1 min window size for data collected at 10 Hz frequency would cover 10 × 60 × 1 = 600 data instances. Suppose that the collected data contains n total instances and k is the number of instances covered in the defined window size (like 600 in the previous example), then at any instance level i, the equation developed can be expressed as follows.

(2) Xi=f(xj) for j= {i to i+k2 when i<ki−k2 to i+k2 when k≤i≤n−ki−k2 to i when i>n−k

where

i = instance level at which moving window concept is applied.
$X_{i}\;$ = computed value after the mathematical operation is applied.
j = range of values covered by the window.
$x_{j}$ = values on which the mathematical operation is applied.
k = number of data instances covered by a defined window size.
n = total number of data instances in the collected dataset.
$f\left(x_{j}\right)$ = mathematical operation.

The mathematical operation can be average, maximum, skewness, *etc.*, depending on the feature to be extracted. The size of moving window used is discussed in Section 2.5.

### 2.4. Classification Algorithm

As mentioned previously in the Section 1, random forest is shown to work better as compared to other algorithms. Consequently, only random forest was used in the present study for the purpose of travel mode classification. 

**Figure 9:**
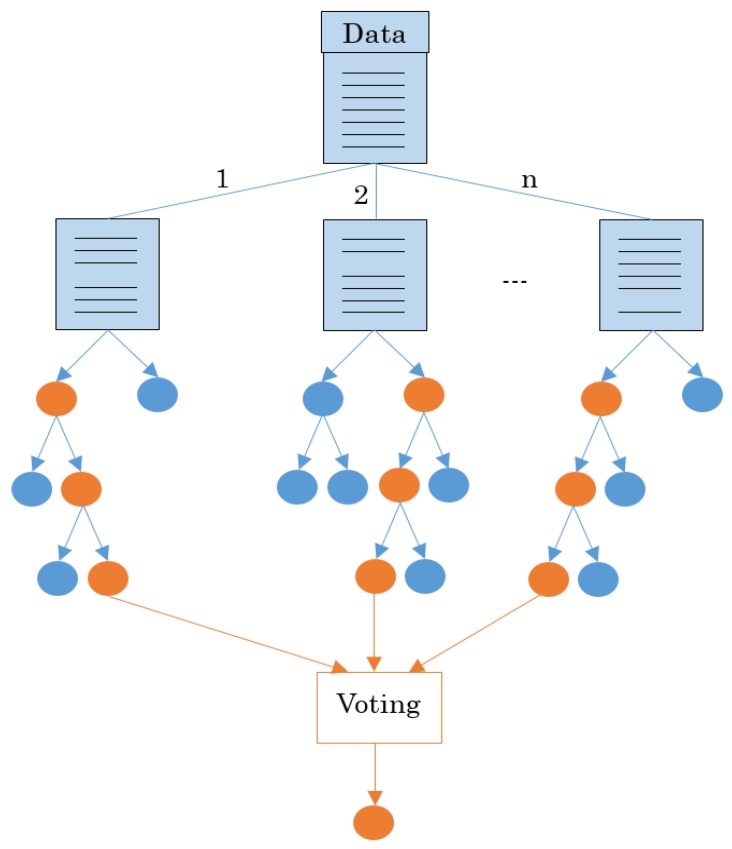
General procedure of Random Forest.

Random forest [59] is an ensemble of decision trees such that each tree is grown independently using a randomly selected dataset while the distribution remains same for all the trees in the forest. As the number of trees in the forest becomes large, the generalization error converges to a limit. The generalization error is dependent on the strength of individual trees and their correlation. Each node within the trees is split using a randomly selected set of features. This randomness introduces robustness to the algorithm against noise. Internal estimates of the algorithm can monitor error, strength and correlation. Variable importance measurement can also be done. Random forest is equally applicable to both classification and regression problems. A general structure of random forest is shown in Figure 9.

R package named “RandomForest” was used in the current study. The package, developed by Liaw and Wiener, use the original coding of the algorithm written by Breiman and Cutler in Fortran, which is imported into R environment. It has the ability to combine various ensembles of trees, extract a single tree from a forest, add trees to an ensemble, extract variable importance measures, *etc.*; and, of course, includes the training of algorithm, predicting and plotting the results. Default values were used for various variables involved in the algorithm whereas the number of trees to be grown was set to 100. From our previous studies, it has been learned that 100 trees are enough for such kind of classification and increasing the number will not be helpful. The number was further confirmed for the data used in the current study. 

The classification results reported in this study are in the form of producer accuracy. For example, if the accuracy is reported to be 70%, it means that 70% of the data belonging to a certain known travel mode (ground truth) is classified correctly as that particular mode by the algorithm. In other words, for any mode “a”

(3) $$Accuracy=\frac{number\;of\;instances\;correctly\;classified\;as\;mode\;\prime \prime a\prime \prime \;by\;algorithm}{total\;number\;of\;instances\;belonging\;to\;mode\;\prime \prime a\prime \prime }$$

### 2.5. Moving Window Size

**Figure 10:**
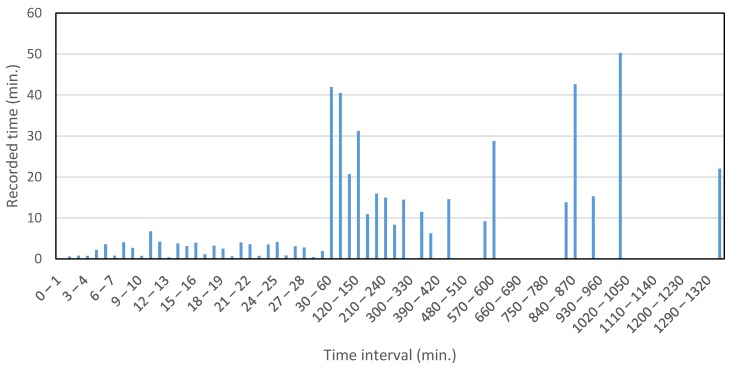
Distribution of trips according to travel time.

According to a previous study [60], the average commute travel time for walking is 16.15 min. Walking is generally the travel mode for the shortest trips; the moving window size should therefore be less than the average value of 16.15 min. 10 min moving window size was selected. Although some trips will even be shorter than 10 min, as is also evident from the distribution of collected data with respect to time intervals shown in Figure 10 (3.37% of the collected data were less than 10 min), the window size cannot be reduced to cover all the trips because then the moving window concept will be useless. 

The aim of moving window is to smoothen the data, and hence decreasing the variation range; this goal cannot be achieved if a very small window size is utilized. In Figure 10, the total recorded time for each trip falling into the various time interval slots (x-axis) was added and plotted on the y-axis.

### 2.6. Data Frequency

As already mentioned in Section 2.1, the data collection frequency varied from 12 to 16 Hz, therefore to attain a uniform frequency, the data were scaled down to 10 Hz. This was achieved by cumulating the time intervals between successive readings. As the sum of time intervals exceeded 0.1 s, the corresponding acceleration reading was selected and the cumulative sum was reset to zero in order to proceed further. In this manner, all data were screened and the readings spaced at 0.1 s apart were selected. The process can be further understood by an example provided in Table 4. It can be observed that the time interval is cumulated until it exceeds the required data interval (2 s or 0.5 Hz in this case), after which it is reset to zero and the corresponding acceleration reading is picked up. The process is repeated for the subsequent readings till all recorded data are scanned. The same procedure was repeated to attain datasets for reduced frequencies: 4 Hz (0.25 s), 2 Hz (0.5 s), 1 Hz (1 s), 0.5 Hz (2 s), 0.33 Hz (3 s), 0.25 Hz (4 s) and 0.2 Hz (5 s). The window size taken was 10 min, so depending on the various data collection frequencies (10 Hz or 1 Hz or any other value), the number of readings in each window will differ, e.g., for 10 Hz, the window will cover 600 readings, whereas for 1 Hz, the window will cover 60 readings.

### 2.7. Amount of Learning Data

**Figure 11:**
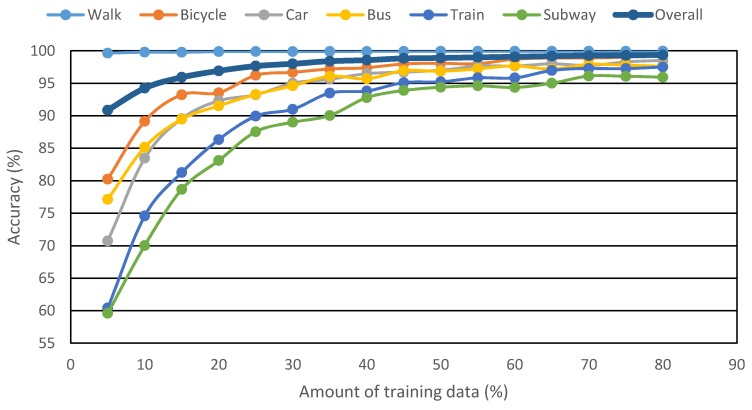
Change in classification accuracy with amount of training data.

For applying the classification algorithm, some portion of the total collected data should be used to train the classifier. Different values of learning data have been used by researchers (Table 5). It is evident from the table that no single value has been agreed upon by researchers. Moreover, the values listed in the table were selected arbitrarily, without any empirical support. For the current study, learning data percentages varying from 5% to 80% were tested on 0.2 Hz data (Figure 11). 

The lowest frequency value was selected as it was expected that the accuracy will be lower as compared to other frequencies and hence the accuracy variation with respect to amount of learning data will be more visible. Regarding the development of learning dataset, stratified random sampling was employed, wherein equal percentage data from each mode was randomly selected.

The results exhibited in Figure 11 are quite logical, with the accuracy increasing with increase in the share of learning data. It is evident that, except walk, all other modes show deteriorated accuracy as the learning data share is reduced. This trend might be specific to the data used in this study because of the huge share of walk instances. The figure suggests that better prediction results can be achieved by simply increasing the amount of learning data compared to the test data. However, this means that for the deployment of the developed methodology for real data, the requirement of a huge controlled survey for obtaining the learning dataset is essential. This requirement will limit the applicability of the approach; therefore, a methodology should be developed that will utilize comparatively fewer learning data but at the same time provide acceptable prediction accuracy.

From Table 6, which is the quantitative translation of Figure 11, it can be seen that increasing the learning data from 5% to 10%, the prediction accuracy increased by about 3.4%, but from 10% to 80% the increase is only 5%. In other words, the prediction accuracy decreased steadily until 10% learning data, after which a drastic drop was witnessed. It was therefore decided to set the amount of learning data to 10%.

## 3. Results and Discussion

Using 10 min moving window to extract the features and 10% data to train the algorithm, classification results were computed for datasets with varying recording frequencies. Additionally, the computation times were also recorded for each dataset, in order to aid in the comparison. Table 7 gives the overall results along with the computation times and Table 8 provides the detailed results in the form of confusion matrices. It is evident from Table 7 that the overall classification accuracy decreases with decrease in data frequency. 

It is already established from Table 6 that the accuracy increases with increase in amount of training data. The trend observed in Table 8 might also have the same reason. With increase in frequency, the amount of data also increased, which in turn increased the training data. Moreover, moving window concept seems to extract better feature values for high frequencies, as the outliers are averaged over a wider range, hence reducing their impacts. The other criterion observed is the time spent in computation. The computation time depends on the amount of data and as the data decreases with the decreased frequency, even though the recorded total time remains the same, the time required for computing decreases. Thus, if the required classification accuracy is more than 99%, then 1 Hz frequency will meet that condition with a 94% decrease in computation time compared to 10 Hz, while the difference in accuracy would be only 0.8%. Furthermore, as mentioned in Section 3, the power consumption will also be reduced. 

Hence, selection of data collection frequency is very crucial, as it not only controls the classification accuracy but also the efficiency of the methodology. Nevertheless, there is a tradeoff between the accuracy and efficiency of the methodology. Therefore, researchers should select the frequency according to their specific needs. Table 9 provides an insight into the prediction accuracy for 0.2 Hz frequency data, with respect to entire trips. One thing to note here is the slightly larger number of trips (625) than reported in Table 3 (559). This is due to breaking up of larger trips into multiple smaller ones when 60 s dwell time was used for trip segregation.

**Figure 12:**
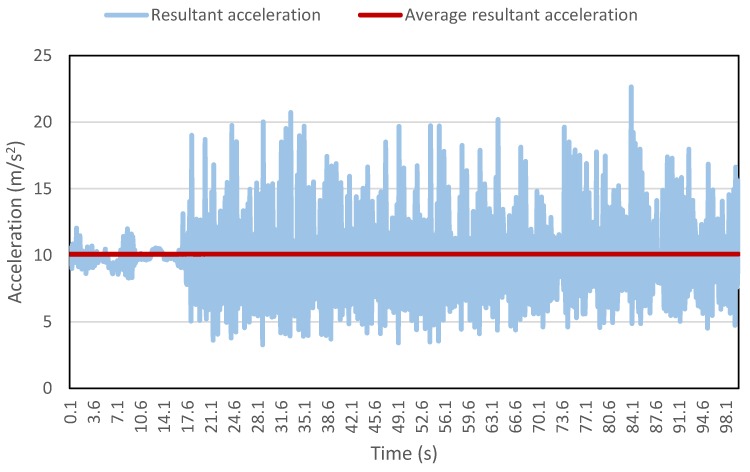
Resultant acceleration and average resultant acceleration for part of a walking trip.

A valid question arises as to the reason for the remarkably high detection accuracy by this methodology. The secret lies in the moving window concept used to extract the various features. Figure 12 shows the resultant acceleration data collected for a part of a walking trip. The average resultant acceleration calculated by moving window is also shown in the figure. It is evident that the average values approximately remain constant, hence providing a very useful feature for the algorithm. If the algorithm is trained using only a few average values, then the algorithm will very easily identify the remaining values against the values from other modes. Moreover, additional features like maximum resultant acceleration, standard deviation, skewness and kurtosis refine the classification process and decrease the number of misclassifications. Conventionally, researchers use specific time windows, mostly having 50% overlap, to extract various features [21,29,47,54,62]. One of the problems with this kind of approach is the loss of data points. For example, for data collected at one reading per second (1 Hz) and a time window of 10 s with 50% overlap, the extracted features will have a frequency of one reading per 5 s (0.2 Hz).

**Figure 13:**
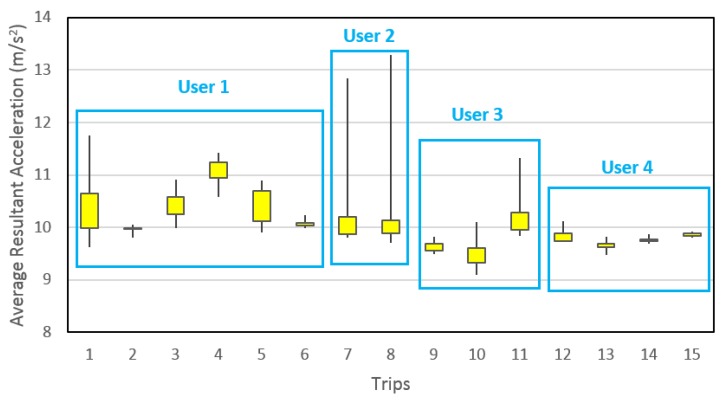
Convergence of data due to big window size.

This explains why moving window was used but does not justify the large window size selected, which might result in excessive overlapping and consequently high prediction accuracies. The reason for using this approach lies in the real world application design of the developed methodology. Generally, people have unique walking and driving patterns, even if they usually stick to a distinctive routine while commuting daily via public transportation. To predict the mode of transportation of a person by studying a completely different person might not yield better results. However, if the prediction is done by studying limited data yielded from the same person, the accuracy will certainly be much better. As the algorithm requires training data, the application design is such that the participants will be asked to at least annotate one day’s data (encouraged by providing some incentive like free cinema tickets, gift vouchers, *etc.*), all of which will be regarded as the training data. After that, the participants just need to keep the application running in the background for the intended period of the survey. In such a design, the big window size does not pose a problem; in fact, it helps to achieve higher prediction accuracy by smoothening the data and bringing it near to the training data. To explain this, Figure 13 demonstrates the average resultant acceleration values, calculated by a 10 min moving window, for first day walking trips made by four participants only. It is evident from the figure that large window size brings the average resultant acceleration data for each trip, by a particular participant, closer to an average value. Hence, it allows the correct prediction within each participant’s data. Note that the figure shows only one feature. When assisted with a number of other features, the prediction process becomes efficient. This is the probable reason behind the extraordinarily high detection accuracies achieved in this study. To include randomness into the present analysis, the training data were randomly selected rather than taking entire trips. The aim is to assist the travel data collection survey; therefore, the predictions need not to be in real-time. Needless to say, it can be used for real-time prediction but then the window size should be decreased so as to abstain from unnecessarily long lag. The grouping of data in Figure 13 should not be confused with window size, which remained constant throughout the entire data for the calculation of all features. This grouping is merely to assist in understanding the advantage of using the large window size of 10 min. Furthermore, each trip demonstrates the spread of average resultant acceleration values calculated using 10-min window size.

**Figure 14:**
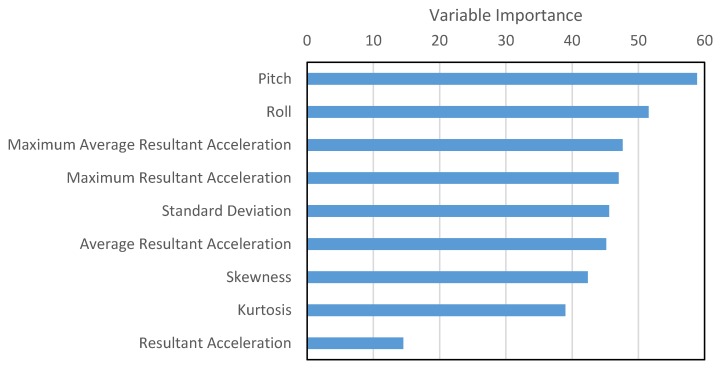
Variable Importance.

The variable importance, calculated by random forest, is shown in Figure 14. It is evident that all features, including orientation readings, are important and add to the predictive power of the algorithm. Resultant acceleration is least important, possibly because all other features are extracted from it and within the extracted features the distinguishable information is magnified. Resultant acceleration can therefore be eliminated from the list of features.

## 4. Conclusions and Future Work

Smartphones are opening up a new horizon for introduction of technology to solve problems in the transportation sector. Travel data collection method can be revolutionized by employing smartphones for passive data recording. This vast possibility is identified by researchers all over the world and much research is being undertaken. The present study is expected to contribute to the ongoing research. The developed methodology takes the data from smartphone sensors as the input information. All this input data can be passively recorded without any effort required on the part of the smartphone carriers. 

The current study demonstrated that data recording frequency has huge impacts on the accuracy and efficiency of the methodology. The frequency should be selected with care, as the accuracy decreases with decrease in frequency but simultaneously, the time required for computation also drops. As computation cost will play a decisive role for huge amounts of data, when data collection by smartphones will be applied on a large extent, selection of suitable frequency value will become all the more important. The researcher has to settle for a compromise between accuracy and computational cost. The results showed that an impressive overall classification accuracy of 99.96% can be achieved, with identification level of no mode less than 99.8%. The main sensor value used to extract further features was the magnitude of resultant acceleration. As individual accelerations are affected by activities performed on the smartphones, it is likely that the calculated magnitude will be slightly different for the same mode among smartphones in use and not in use. This in turn will influence the extracted features. It is therefore necessary to investigate this variability and its effect on mode detection. 

Initially, automatic mode detection will complement the traditional travel data collection methods by providing accurate and detailed travel information. The participants will no longer need to keep a mental note of where and when they took a trip. All this information will be provided by their smartphones, and the accuracy will obviously be higher. The final form of smart data collection would be making the traditional methods redundant. In future, the smartphone will not only be able to determine the mode of transportation used but will also be able to identify the family, thereby extracting the family data from governmental records like number of family members, their ages, salaries, *etc.* Moreover, by interacting with nearby smartphones, the identity of the accompanying persons will also be ascertained. We are moving briskly towards that era, with ever increasing smartphone penetration as well as tremendous increase in Internet access. 

The sharp decrease in accuracy below 10% learning data, as mentioned in Section 2.7 might also be the result of small amount of collected data. As the amount of training data are increased, the algorithm becomes more and more intelligent towards predicting unknown examples correctly, until a certain amount is achieved, after which additional training examples do not add substantial detection power to the algorithm. In other words, the algorithm is fully trained and can predict huge amounts of unknown examples. Future studies should keep this aspect in mind and, while using large dataset, report the training data in terms of data points or number of trips rather than percentage of total data. Furthermore, the saturation point should be determined to decide the amount of training data. 

One of the major limitations of this study is trip segmentation. Trip segmentation is implicitly added to the data by deleting sensor data during stay or periods of non-activity. It is then coupled with 60 s dwell time to divide the data into trips. In reality, the analyst will be unaware of breaks in the data; therefore, an efficient trip segmentation methodology should be developed. Another major constraint is the unequal representation of various modes in the collected data. Although the data provide a realistic picture of typical Japanese lifestyle, where walking has a major share in daily travelling, this may overshadow other modes. It can be witnessed from the mode-wise classification results, where walk showed outstanding accuracy as compared to other modes. Moreover, due to the massive amount of walk data used for training the algorithm, other modes are predominantly misclassified as walk. Applying the developed methodology for data with comparable representation from all modes might yield different results and should therefore be tested. Another limitation is the small amount of data used in the study. More data should be tested so that the developed methodology may obtain wider acceptance. Effort should be made in order to further decrease the percentage of data used to train the algorithm while attaining similar accuracy levels. This will ensure accurate data interpretation for a large amount of data collected, even when using a small percentage for training purpose. Moreover, variation in data and classification accuracy among different users should be explored to understand the role of users. This may provide new ideas to tackle the issue at hand.

## Figures and Tables

**Table 1:** Example of retained and discarded data.

Trip Confirmed by Participant	GPS Coordinates	Acceleration (m/s^2^)	Remarks
Trip Start	34.6707318, 135.1759662	10.01443	Retained
Driving	34.6707316, 135.1759678	9.836915	Retained
	0, 0	9.505924	Discarded
	34.6707313, 135.1759697	9.253243	Retained
Trip End	34.670731, 135.1759717	9.579927	Retained
Unlinked Data	34.6707307, 135.1759734	10.13853	Discarded
	34.6707306, 135.1759744	10.04357	Discarded

**Table 2:** Details of participants.

Gender	Number	Average Age	Number by Age					
			20–30	30–40	40–50	50–60	60–70	Above 70
Male	27	35.56	7	10	4	1	4	1
Female	23	32.17	6	8	7	2	0	0

**Table 3:** Amount of data recorded by smartphones.

Mode	No. of Trips	Total Time (Hours)	Amount of Data Instances	Percentage of Total Data
Walk	442	144	5,186,095	71%
Bicycle	10	9	326,500	4%
Car	31	14	500,410	7%
Bus	21	11	381,698	5%
Train	45	18	659,528	9%
Subway	10	7	236,738	3%
Total	559	203	7,290,969	99%

**Table 4:** Methodology employed for data frequency reduction.

Sr. No.	Time Interval	Acceleration (m/s^2^)	Cumulative Time for 2 s Interval	Acceleration at 0.5 Hz (m/s^2^)
1	0	10.01443	0	10.01443
2	0.6	9.836915	0.6	-
3	0.5	9.505924	1.1	-
4	0.4	9.253243	1.5	-
5	0.6	9.579927	0	9.579927
6	0.6	10.13853	0.6	-
7	0.4	10.04357	1	-
8	0.5	10.01443	1.5	-
9	0.4	9.836915	1.9	-
10	0.5	9.505924	0	9.505924

**Table 5:** Percentage of learning data used by various researchers.

Study	Percentage of Data Used for Training (%)
Nick, Coersmeier [47]	90
Tragopoulou, Varlamis [61]	80
Lester, Choudhury [45], Nitsche, Widhalm [62]	75
Nham, Siangliulue [29]	70
Abdulazim, Abdelgawad [32]	65
Figo, Diniz [48]	50

**Table 6:** Increase in accuracy with amount of training data.

Amount of Training Data (%)	Overall Accuracy (%)	Increase in Accuracy (%)
5	90.863	-
10	94.253	3.390
15	95.928	1.674
20	96.902	0.974
25	97.653	0.751
30	97.995	0.342
35	98.401	0.406
40	98.566	0.165
45	98.835	0.269
50	98.887	0.053
55	99.022	0.135
60	99.059	0.037
65	99.190	0.131
70	99.271	0.081
75	99.304	0.033
80	99.356	0.052

**Table 7:** Summary of classification results.

Results	Data Frequency (Hz)							
	10	4	2	1	0.5	0.33	0.25	0.2
Training instances	729,097	364,588	184,297	98,301	50,805	34,291	25,527	20,566
Test instances	6,561,871	3,281,290	1,658,673	884,709	457,244	308,623	229,746	185,092
Overall accuracy (%)	99.963	99.883	99.690	99.131	98.012	96.731	95.415	94.477
Time (s)	304.86	102.09	42.05	19.37	9.45	6.02	5.35	3.53

**Table 8:** Detailed classification results.

Data Frequency (Hz)	Actual Class	Predicted Class						Ground Truth	Accuracy (%)
		Walk	Bicycle	Car	Bus	Train	Subway		
10	Walk	4,667,281	40	51	39	34	40	4,667,485	99.996
	Bicycle	104	293,731	2	1	12	0	293,850	99.960
	Car	543	0	449,816	6	3	1	450,369	99.877
	Bus	212	0	3	343,310	1	2	343,528	99.937
	Train	796	5	2	7	592,755	10	593,575	99.862
	Subway	460	0	7	31	15	212,551	213,064	99.759
4	Walk	2,336,253	36	63	40	62	76	2,336,530	99.988
	Bicycle	173	146,767	2	2	0	0	146,944	99.880
	Car	618	10	224,715	5	8	1	225,357	99.715
	Bus	379	0	36	171,462	19	9	171,905	99.742
	Train	1386	1	24	17	295,489	2	296,919	99.518
	Subway	825	0	21	10	28	102,751	103,635	99.147
2	Walk	1,183,188	29	38	8	71	49	1,183,383	99.984
	Bicycle	364	73,251	7	1	9	0	73,632	99.483
	Car	1068	5	111,905	4	71	14	113,067	98.972
	Bus	732	0	13	85,525	8	0	86,278	99.127
	Train	1775	1	15	8	147,214	0	149,013	98.793
	Subway	833	8	1	6	8	52,444	53,300	98.394
1	Walk	631,319	45	61	29	61	56	631,571	99.960
	Bicycle	371	38,863	6	0	10	0	39,250	99.014
	Car	1302	6	58,898	6	26	4	60,242	97.769
	Bus	1184	19	50	44,736	5	9	46,003	97.246
	Train	2797	3	21	1	76,555	20	79,397	96.421
	Subway	1497	2	22	60	16	26,649	28,246	94.346
0.5	Walk	326,154	50	93	84	72	26	326,479	99.900
	Bicycle	565	19,691	41	1	7	0	20,305	96.976
	Car	1536	0	29,598	5	28	0	31,167	94.966
	Bus	1325	1	61	22,359	11	3	23,760	94.104
	Train	3546	6	33	5	37,454	22	41,066	91.204
	Subway	1527	0	17	19	5	12,899	14,467	89.162
0.33	Walk	220,111	57	88	47	80	27	220,410	99.864
	Bicycle	547	13,133	10	1	7	0	13,698	95.875
	Car	1936	6	19,039	10	37	2	21,030	90.533
	Bus	1254	34	38	14,658	37	6	16,027	91.458
	Train	3813	40	55	15	23,770	17	27,710	85.781
	Subway	1881	2	15	17	9	7824	9748	80.263
0.25	Walk	163,812	102	93	53	73	44	164,177	99.778
	Bicycle	753	9396	9	0	4	0	10,162	92.462
	Car	1928	0	13,648	3	35	0	15,614	87.409
	Bus	1670	3	64	10,135	36	7	11,915	85.061
	Train	3841	15	19	19	16,669	11	20,574	81.020
	Subway	1709	24	2	9	8	5552	7304	76.013
0.2	Walk	131,946	65	96	102	34	32	132,275	99.751
	Bicycle	742	7431	5	7	3	0	8188	90.755
	Car	2154	5	10,398	5	19	1	12,582	82.642
	Bus	1186	4	49	8319	22	19	9599	86.665
	Train	3908	22	68	3	12,579	0	16,580	75.869
	Subway	1608	3	3	32	26	4196	5868	71.506

**Table 9:** Classification results of trips for 0.2 Hz data.

Prediction Accuracy (%)	No. of Trips
100	453
95–100	59
90–95	16
85–90	15
80–85	8
75–80	10
70–75	12
0–70	52
Total	625
